# Durasphere^®^ EXP: a non-biodegradable agent for treatment of primary Vesico-Ureteral reflux in children

**DOI:** 10.1590/S1677-5538.IBJU.2017.0514

**Published:** 2018

**Authors:** Unsal Ozkuvanci, Muhammet Irfan Donmez, Faruk Ozgor, Akif Erbin, Özge Pasin, Ahmet Yaser Muslumanoglu

**Affiliations:** 1 Department of Urology Haseki Training and Research Hospital, Fatih, Istanbul, Turkey; 2 Department of Urology, Istanbul Faculty of Medicine, Istanbul University, Fatih, Istanbul, Turkey; 3 Department of Biostatistics, Istanbul Faculty of Medicine, Istanbul University, Fatih, Istanbul, Turkey

**Keywords:** Durasphere [Supplementary Concept], Vesico-Ureteral Reflux, Child

## Abstract

**Introduction:**

Durasphere^®^ EXP (DEXP) is a compound of biocompatible and non--biodegradable particles of zirconium oxide covered with pyrolytic carbon. The aim of this study is to evaluate the durability of off-label use of DEXP in the treatment of primary vesicoureteral reflux in children.

**Materials and Methods:**

Patients who underwent subureteric injection of DEXP for the correction of primary VUR were retrospectively reviewed. Patients aged >18 years as well as those who had grade-I or -V VUR, anatomic abnormalities (duplicated system, hutch diverticulum), neurogenic bladder or treatment refractory voiding dysfunction were excluded. Radiologic success was defined as the resolution of VUR at the 3rd month control. Success was radiographically evaluated at the end of the first year.

**Results:**

Thirty-eight patients (9 boys, 29 girls; mean age, 6.3±2.7 years) formed the study cohort. Forty-six renal units received DEXP (grade II: 22; grade III: 18; grade IV: 6). Mean volume per ureteric orifice to obtain the mound was 0.70±0.16mL. First con- trol VCUG was done after 3 months in all patients. After the first VCUG, 6 patients had VUR recurrence. Short-term radiologic success of DEXP was 84.2%. Rate of radiologic success at the end of the first year was 69.4% (25/32). Lower age (p:0.006) and lower amount of injected material (p:0.05) were associated with higher success rates at the end of 1 year.

**Conclusion:**

This is the first study to assess the outcomes of DEXP for treatment of primary VUR in children. After 1 year of follow-up, DEXP had a 69.4% success rate.

## INTRODUCTION

Vesicoureteral reflux (VUR) is a challenging problem in pediatric urology. Treatment options include close observation, continuous antibiotic prophylaxis, endoscopic subureteric injection, and open/robotic ureteric re-implantation. Urinary tract infections (UTIs) concomitant with VUR can lead to renal scarring and hence hypertension and renal failure (1). Treatment aim is to prevent febrile UTIs.

Subureteric injection of bulking agents has been shown to be a good alternative to ureteric re-implantation. The success of endoscopic treatment is dependent not only to the features of the bulking agent but also grade of VUR, presence of lower urinary tract dysfunction (LUTD) and anatomical abnormalities. The initial substance to be used was Teflon™ while dextranomer/hyaluronic acid (Dx/HA) was the first agent to be approved by the US Food and Drug Administration (FDA) for VUR treatment in children in 2001. Since then, Dx/HA has been used widely for the correction of low-grade VUR and has become the “gold standard”. The migration risk of Teflon, reduced prevalence of overall success (77%) and a relatively high prevalence of recurrence of Dx/HA (11-26%) have had pivotal roles in the development of new bulking agents with better outcomes (2). Subsequently, several non-biode-gradable agents (carbon-coated particles of zirconium oxide, polyacrylamide hydrogel (PAHG), polyacrylate/polyalcohol copolymer (PPC)) have been released on the market. Long-term durability of those materials used in subureteric injection is linked to their non-biodegradability and formation of fibrotic capsules (3).

Another non-biodegradable agent, Durasphere^®^ EXP (DEXP) has been actively used in urological practice since 1999 to treat stress urinary incontinence in women. Here, we evaluated the durability of off-label use of Durasphere^®^ EXP (DEXP) for the treatment of primary VUR in children.

## MATERIALS AND METHODS

The injectable bulking agent DEXP (Carbon Medical Technologies, St. Paul, MN, USA) is a compound of biocompatible and non-biodegrad-able particles of zirconium oxide covered with pyrolytic carbon suspended in a water gel with 2.8% beta-glucan (4). It is a bulking agent used (off-label) for VUR correction in children. Furthermore, it has been used in our daily practice between 2008 and 2013.

**Figure 1:**
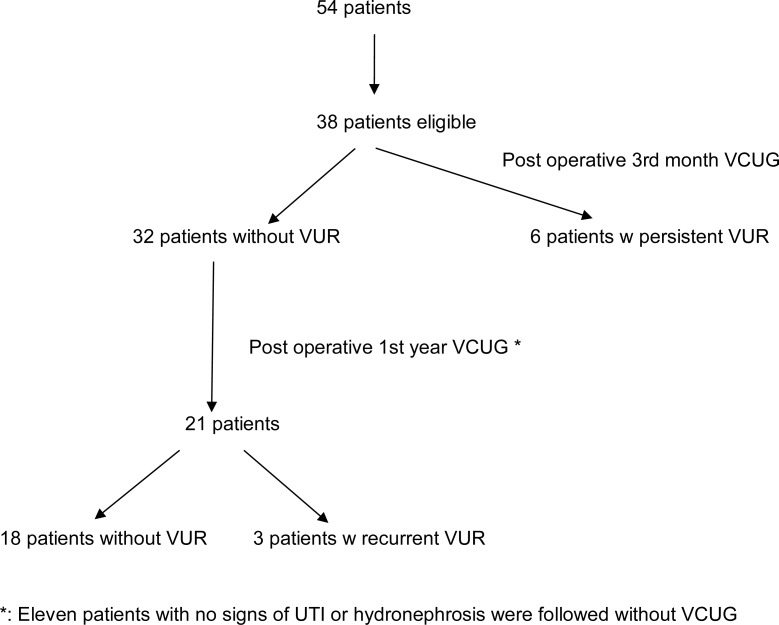
Flow diagram of patients.

After obtaining local review board approval, medical files of patients who had undergone subureteric injection of DEXP for correction of primary VUR in our clinic between February 2008 and March 2013 were reviewed retrospectively. Age, sex, presenting symptoms, laterality and degree of VUR (classification set by the International Reflux Study Committee), presence of renal scars in DMSA (Di-Mercapto-Succinic Acid) scintigraphy, volume of injected material, and previous intervention for VUR were noted. Patients aged >18 years as well as those who had VUR grade I or V, anatomic abnormalities (duplicated system, hutch diverticulum), neurogenic bladder or voiding dysfunction refractory to appropriate medical treatment were excluded (Figure-1). All patients had written informed consent prior to surgery.

Endoscopic procedures were performed after a sterile urine culture was obtained in all patients. Subureteric injection was undertaken under general anesthesia using a 9.5-Fr, 6° pediatric cystoscope (Karl Storz Endoscopy, Slough, UK) with a metal 3.7-channel, 20-G, pencil point-tip needle (254mm or 381mm) by two surgeons (A.Y.M., U.O.). DEXP was applied using the STING/hydrodistention method. All patients were discharged after spontaneous voiding on the day of the procedure. Patients were continued on their previously started antibiotic prophylaxis until the first voiding cystourethrogram (VCUG) at 3-month follow-up. Radiologic success was defined as the resolution of VUR at the 3rd month control VCUG. The durability of success in those patients was ra-diographically and clinically evaluated at the end of the first year.

### Statistical analysis

The normality hypothesis of the variables was examined by the Shapiro Wilk test. The group comparisons between the categorical variables were analyzed using Kruskall Wallis test and Dunn test was applied for subsequent multiple comparisons (Post-hoc). The comparison of the qualitative variables was done using the Fisher Freeman-Halton test. Statistical analyzes were performed using SPSS Version 21 software.

## RESULTS

Thirty-eight patients (9 boys, 29 girls; mean age, 6.3±2.7 years) and 46 renal units were treated with DEXP. Indications for subureteric injection were recurrent febrile UTI in 25 patients, VUR and renal scarring (in DMSA scintigraphy) in 10 patients, and non-resolving VUR at the follow-up for antenatal hydronephrosis in 3 patients. VUR was unilateral (13 right, 17 left) in 30 patients and bilateral in 8 patients. Ten patients had received two subureteric injections previously using Dx/HA, whereas 28 received their first subureteric injection. A total of forty-six renal units received DEXP (grade II: 22; grade III: 18; grade IV: 6). Mean volume per ureteric orifice in order to ob-tain the mound was 0.70±0.16mL. First control VCUG was undertaken after 3 months in all pa-tients. After the first VCUG, 6 patients (2 bilateral, 4 unilateral disease previously) had failure. Thus, the 3-month radiologic success rate was 84.2% (Table-1).

**Table 1:** Results of injection of Duraspehere^®^ EXP according to grades of vesicoureteral reflux.

Preoperative VUR	VCUG 3 months after surgery	VCUG 1 year after surgery
(n: Renal Unit)	(VUR/no VUR Renal Unit) Success (%)	(VUR/no VUR Renal Unit) Success (%)
Grade-II VUR	2/20	2/10
n=22	90.9%	83.3%
Grade-III VUR	4/14	2/4
n=18	77.7%	66.6%
Grade-IV VUR	2/4	1/1
n=6	66.6%	50%

During the follow-up of the patients with no VUR at 3-months, 1 patient had febrile UTI. Imminent VCUG revealed VUR recurrence (pretreatment unilateral grade-IV VUR, one patient was lost to follow-up). First-year control VCUG showed that 4 out of 29 patients had VUR recurrence. Of those with recurrent VUR, one patient had bilateral grade-III VUR and renal scarring preoperatively. The grade of VUR was down graded to bilateral grade 2. Also, 1 had bilateral grade-III VUR, 1 had unilateral grade-III VUR and 1 had unilateral grade IV VUR, previously. However, all of those patients were infection and LUTD free. Radiologic success at the end of the first year was 69.4%.

Nine patients (9 renal units) without VCUG at 1 year were accepted as clinically successful since they were UTI and LUTD free. Success at 1st year was higher in the patients with lower age (5.64 vs. 9.66, p:0.006) and less amount of injected material (0.66 vs. 0.75, p: 0.05).

Moreover, presence of renal scar and presentation with recurrent UTI were the risk factors for reduced success rate (both p:0.023). The ratios and significance of variables are given in Table-2.

**Table 2:** Ratio comparison of categorical and continuous variables (Fisher Fremann-Halton test).

		No VUR at 3rd and 12th months (%)	No VUR at 3rd month but VUR at 12th month (%)	p value
**Gender**				
	Male	29	0	0.3
	Female	71	100	
**Type of presentation**				
	UTI	67	16.7	
	VUR + Renal scar	29	50	**0.023**
	Antenatal hydronephrosis f/u	3.2	33.3	
**Laterality**				
	Right	38.7	50	0.49
	Left	61.3	50	
**Degree of VUR**				
	Grade II	58	33.3	0.29
	Grade III	35.5	33.3	
	Grade IV	6.5	33.3	
**Presence of renal scar**				
	No	71	16.7	**0.023**
	Yes	29	83.3	
Age (years)		5.6	9.6	**0.006**
Injected volume (mL)		0.66	0.75	**0.05**

After surgery, 1 patient had a transient de novo hydronephrosis without functional loss according to scintigraphy. In addition, ten patients had a mean follow-up of >3 years without any sign of a UTI or urinary obstruction.

## DISCUSSION

Biodegradable synthetic materials have a high rate of reabsorption over time. Dx/HA is included in this group, and its rate of recurrence in long-term follow-up studies has been reported to be 11-26% (5). It has been suggested that non-biodegradable synthetic materials become more persistent by forming a fibrotic capsule. Such materials available currently are carbon-coated particles of zirconium oxide, PAHG and PPC.

Durasphere^®^ was introduced as a biocompatible, non-migrating, non-erosive, non-immunogenic, non-biodegradable substance comprising large (212-500 microns) carbon-coated particles of zirconium oxide. In 1999, it was approved by the US FDA for the treatment of stress-type urinary incontinence caused by sphincter insufficiency in women. Its production was discontinued because of its high viscosity (which caused difficulties in injection) and because patients had asymptomatic migration of lymph and formation of sterile pseudo-abscesses after periurethral injection. The manufacturer replaced the product with a sub-stance of smaller-sized particles (90-212 microns) under the name “Durasphere^®^ EXP” (6).

Some authors have linked these unwanted complications of DEXP with injection of excessive amounts of DEXP into the periurethral region as well as injection of DEXP into blood vessels (7). Prevalence of formation of sterile pseudo-abscesses in transurethral injection of Dx/HA has been reported to be 16% (8). It is not known if this new product causes migration or pseudo-abscess formation if used in smaller amounts.

In a study comparing the persistency of Durasphere^®^ with collagen in women with urinary incontinence, long-term results were better for Durasphere^®^ than for collagen (9). However, the effect of Durasphere^®^ was reduced over time. How the effect persists over time in VUR treatment is not known.

Several studies have evaluated the use of non-biodegradable substances in VUR treatment. However, the number of studies focusing on off-label use of DEXP in VUR treatment is small. In our country, off-label use of DEXP has been for the treatment of VUR in children since 2006 (10).

In 2011, it was reported that the prevalence of clinical success of DEXP for treatment of recurrent transplant pyelonephritis secondary to VUR to the transplant kidney in 8 patients was 75% during a period of follow-up of 3-52 months, but the of long-term efficacy of DEXP treatment was not reported (11).

Evaluation of success of endoscopic treatment can be done by clinical (urine culture) and radiologic (VCUG, ultrasonography) means, as well as by scintigraphy. We found the rate of short-term (3 months) radiologic success of DEXP to be 81.3% (32/38). Rate of radiologic success at the end of the first year was 69.4% (25/32). Our results indicated that durability of success is significantly higher in younger children and lower VUR degree. Additionally, achieving the intended amount of mound using as little injection material as possible was found to be associated with long-term success.

The short-term success of non-biode-gradable PAHG for endoscopic treatment of VUR was shown to be 81.2%. The authors found at the second injection that the mound obtained with PAHG at first injection was flattened or displaced, and that the fluidity of PAHG could be the reason for such failures (5). In our study, we observed medially displacement of the material in 2 of 3 recurrent cases.

PPC was shown to have good overall success in VUR treatment (83.6-95%) (12). Recurrent VUR was not reported in 140 patients (106 of these patients had a control VCUG) during a follow-up >3 years (12). Some authors argue that PPC injection carries a great risk of ureterovesical obstruction, and recommended close monitoring after surgery (13).

In our study, de novo hydronephrosis or a UTI were not observed in 10 cases who had a follow-up of 3 years. Postoperatively, 1 patient had a transient de novo hydronephrosis without functional loss according to scintigraphy and the hydronephrosis resolved eventually. The decrease of success in time may be related to the substance itself as well as patient related factors (undiagnosed bladder dysfunction, lack of muscular support at the level of ureteral hiatus). This finding confirms that biodegradability may not be the scapegoat for reflux recurrence after endoscopic injection.

One important aspect of the material was the rapid clotting potential. If the initial puncture was not in the right sub-mucosal plane or the needle faces some resistance, carbon particles may not find their route and the water-based carrier gel component of the material goes through the needle. If that happens, the carbon particles would no longer flow through the needle that ne-cessitate a brand-new needle and injection material to be used.

Our study was limited due to its retrospective nature. In addition, the prevalence and type of complications were not reported. The sustainability of the ureteric mound could have been measured during control urinary ultrasonography.

## CONCLUSIONS

This is the first study on the 1-year outcomes of DEXP for endoscopic treatment for primary VUR in children. DEXP had a durability of 69.4%. Our results reveal that biodegradability of the injected material is not the sole factor for the late recurrence of endoscopic treatment of VUR. Further prospective, randomized controlled trials with long-term results are needed to determine the efficacy and durability of DEXP for VUR treatment.

## COMPETING INTERESTS

Ethical Standard: All procedures performed in studies involving human participants were in accordance with the ethical standards of the institutional and/or national research committee and with the 1964 Helsinki declaration and its later amendments or comparable ethical standards. Formal consents were obtained from parents.
